# Hepatitis E Virus Genotype 3 in Quindío, Colombia: One Health Surveillance and First Genomic Characterization Reveal Cryptic Regional Divergence

**DOI:** 10.3390/tropicalmed11100290

**Published:** 2026-10-13

**Authors:** Maria Mercedes González, Omar David Moreno, Leonardo Padilla-Sanabria, Jhon Carlos Castaño-Osorio

**Affiliations:** GEPAMOL/GYMOL Research Group, Faculty of Health Sciences, Universidad del Quindío, Armenia 630004, Colombia; mmgonzalez@uniquindio.edu.co (M.M.G.); lpadilla@uniquindio.edu.co (L.P.-S.); jhoncarlos@uniquindio.edu.co (J.C.C.-O.)

**Keywords:** hepatitis E virus, genotype 3, one health, Colombia, environmental surveillance, zoonosis, Oxford nanopore, k-mer fingerprinting, genomic divergence, molecular epidemiology

## Abstract

Hepatitis E virus genotype 3 (HEV-3) is a zoonotic pathogen whose burden in Latin America remains underestimated. We conducted a cross-sectional One Health surveillance study (2019–2024) in Quindío, Colombia, testing 42 pig fecal, 8 farm runoff, 4 tributary water samples, and 5 clinical serum samples from patients with hepatitis of undetermined etiology by reverse transcription quantitative PCR (RT-qPCR). Positive and selected clinical/environmental samples underwent Oxford Nanopore MinION sequencing targeting the open reading frame 2 and 3 (ORF2/ORF3) junction, k-mer fingerprinting (k = 25), consensus generation, phylogenetic analysis, and Sanger confirmation. HEV RNA was detected in 8/42 pig fecal samples (19.0%), 4/8 runoff samples (50.0%), 1/4 tributary samples (25.0%), and 4/5 serum samples (80.0%). K-mer analysis identified strong HEV signals in two clinical samples (44.5% and 39.5% of reads) and wastewater (97.2%). Phylogenetic analysis classified Colombian isolates within HEV-3 (bootstrap 83%), while Sanger sequencing confirmed HEV-specific motifs. Two sequences were deposited in GenBank (PZ262436, PZ262437), representing the first Colombian HEV-3 genomic records. These findings demonstrate HEV-3 circulation across porcine, environmental, and human compartments and highlight substantial genomic divergence, supporting locally calibrated molecular approaches and strengthened One Health genomic surveillance in Colombia and Latin America.

## 1. Introduction

Hepatitis E virus (HEV), initially recognized as the agent of enterically transmitted non-A, non-B hepatitis, is now considered one of the most frequent causes of acute viral hepatitis worldwide. The World Health Organization (WHO) estimates approximately 20 million infections and 44,000 deaths annually, disproportionately affecting pregnant women, although most infections are asymptomatic [1]. HEV is classified within the family *Hepeviridae*, subfamily *Orthohepevirinae*; species A (*Paslahepevirus balayani*) comprises the genotypes infecting humans. Genotypes 1 and 2 are restricted to humans and drive large waterborne outbreaks in low-income countries, whereas genotypes 3 and 4 are zoonotic, circulating primarily in swine and causing sporadic autochthonous infections—chiefly through undercooked pork products or direct contact with reservoir animals—in both industrialized and developing nations [2,3].

Environmental circulation of HEV, an established risk factor for waterborne transmission, has been documented mainly in Europe and Asia. In Colombia, the first report of environmental circulation came from Antioquia, where the virus was detected in drinking-water sources and wastewater [4]. Subsequent studies documented HEV-3 in patients with acute hepatitis in Medellín (22.5% by reverse transcription PCR, RT-PCR) [5], seropositivity of 8.7% in Colombian hepatitis patients [6], 4.4% seroprevalence among Quindío residents [7], 15.7% seropositivity among Antioquian pig-farm workers confirming occupational zoonotic exposure [8], and up to 45.2% anti-HEV antibody frequency among rural blood donors in Yarumal, Antioquia [9]. Molecular evidence extended to swine: HEV-3 was found in 41.3% of liver samples from Antioquia abattoirs [10] and 26.9% of porcine fecal samples [11]; this evidence resulted from contrasting Sanger-derived sequences against sequences deposited in GenBank at that time. Despite more than a decade of accumulated serological and molecular evidence spanning environmental, animal, and human compartments, hepatitis E is still not a notifiable disease in Colombia [12,13], and no Colombian HEV sequence had ever been deposited in GenBank or any other international nucleotide database prior to the present study.

This absence is not merely a gap in reporting. A critical, underappreciated obstacle to HEV surveillance in Latin America is the genomic divergence of regional strains from the reference sequences that dominate international databases, which are drawn predominantly from European, Asian, and North American isolates. This divergence directly compromises the sensitivity of alignment-based detection tools—including the Basic Local Alignment Search Tool (BLAST) and reference-trained k-mer classifiers—creating a plausible mechanism for systematic false negatives when regional strains are compared against a poorly representative database [14]. We therefore designed this study with two complementary aims. First, applying a One Health framework, we conducted cross-sectional reverse transcription quantitative PCR (RT-qPCR) surveillance (2019–2024) across environmental, porcine, and human compartments in Quindío department to characterize the extent and distribution of HEV circulation. Second, we applied Oxford Nanopore Technologies (ONT) amplicon sequencing combined with a locally calibrated k-mer fingerprinting pipeline, phylogenetic reconstruction, and Sanger confirmation to directly investigate why standard sequence-based detection had never succeeded for Colombian strains. This combined approach allowed us to both document the One Health circulation of HEV-3 in Quindío and, for the first time, genomically characterize and deposit Colombian HEV-3 sequences in GenBank.

## 2. Materials and Methods

### 2.1. Ethics Statement

The study was approved by the Ethics and Bioethics Committee for Research of the Faculty of Health Sciences, Universidad del Quindío (Acta N° 023, 7 October 2022). The study was also conducted with institutional endorsement from Hospital Departamental Universitario San Juan de Dios del Quindío (Radicado 202201850081131, 18 October 2022).

### 2.2. Study Area

Quindío department is in the Andean region of west-central Colombia (1845 km^2^, population density 306 inhabitants/km^2^), with a substantial agricultural sector including swine farming (seventh largest producer nationally [15]). The study included technified pig farms in six municipalities: Filandia, Circasia, Salento, Calarcá, Montenegro, and Génova (Figure 1). Sampling also included the Barcelona corregimiento of Calarcá; Barcelona is therefore not counted as a separate municipality.

### 2.3. One Health Sample Collection (RT-qPCR Surveillance, 2019–2024)

Between July 2019 and December 2023, we collected pig fecal samples (*n* = 42) from technified swine farms (farrow-to-finish and fattening operations), farm runoff water (*n* = 8), and tributary water (*n* = 4). Fecal samples were processed as a 10% suspension in phosphate-buffered saline (PBS), centrifuged (1500 g/10 min/4 °C), and treated with chloroform (analytical grade, ≥99% purity; 1:1 vol/vol; deproteinization step). Runoff and tributary water samples were collected in sterile polypropylene bottles (1–2 L), maintained at 4 °C, and processed within 24 h of collection, following Báez et al. [4]. Runoff samples were concentrated by the Sobsey method (pH adjustment to 7.0; centrifugation at 3500 rpm/30 min/4 °C; precipitation with 0.3 N NaCl and 8% polyethylene glycol 8000); tributary samples were processed by the two-phase method, a standard approach for environmental viral surveillance [16]. Between 2022 and 2024, five serum samples were additionally obtained from patients hospitalized at a tertiary-care institution in Armenia with a clinical diagnosis of hepatitis of undetermined etiology. RNA was extracted using a commercial kit (alphaClean Pure RNA/DNA Kit, MIKROGEN^®^, Neuried, Germany). HEV genome detection was performed with a WHO-validated commercial RT-qPCR kit (MIKROGEN^®^ ampliCube HEV 2.0 Quant), reported at 100% sensitivity and specificity for HEV species A genotypes 1–4. Each run included manufacturer-provided positive and negative controls. Samples were collected on an opportunity basis; no formal sample size calculation was performed prior to the study.

### 2.4. Molecular Characterization: Sample Selection

A subset of samples—clinical sera confirmed positive by RT-qPCR and one wastewater sample collected in Medellín, Antioquia (~2022) as part of an ongoing regional HEV surveillance effort (Universidad de Antioquia)—underwent deeper molecular characterization to confirm HEV identity independently of the diagnostic kit and to investigate why conventional sequence-based tools had failed to characterize any Colombian strain to date.

### 2.5. RNA Extraction, DNase I Treatment, and cDNA Synthesis

Total nucleic acids were extracted using matrix-appropriate kits: the innuPREP AniCon DNA/RNA kit (Analytik Jena, Jena, Germany) was applied to porcine fecal and environmental water samples, while the RNeasy Mini kit (Qiagen, Hilden, Germany) was used for clinical serum samples. Kits were selected based on matrix type and manufacturer recommendations for the respective sample types; no formal comparative validation between the two kits was performed, which should be considered a study limitation. Prior to cDNA synthesis, extracted RNA was treated with DNase I (New England Biolabs, Ipswich, MA, USA) to remove residual genomic DNA contamination—a step omitted in previous Colombian HEV studies. RNA integrity was preserved with RNaseOUT (Thermo Fisher Scientific, Waltham, MA, USA). cDNA was synthesized using the RevertAid First Strand cDNA Synthesis kit (Thermo Fisher Scientific) with random hexamers.

### 2.6. Amplicon Generation and Oxford Nanopore Sequencing

Amplicons targeting the open reading frame 2 and 3 (ORF2/ORF3) junction (~350–400 bp) were generated by nested RT-PCR using degenerate primers from Rendón et al. (2016) [5]: outer primers HE361 (5′-GCRTGTGGTTTTGGGGCTGAG-3′) and HE364 (5′-GCGRTGGTTTCTGGGGCTGAC-3′), and inner primers HE363 (5′-GMYTTGGTCDGCCAAGHGGA-3′) and HE366 (5′-GYTGGTTCTCAGCCCTTCTGC-3′). Libraries were prepared with the Native Barcoding Kit 24 (SQK-NBD114-24, Oxford Nanopore Technologies, Oxford, UK) and sequenced on a MinION device (Oxford Nanopore Technologies, Oxford, UK) with an R10.4.1 flow cell (FLO-MIN114) for ~48 h. Basecalling used Guppy v7.11.2 (Oxford Nanopore Technologies). Reads were filtered by length (150–450 bp) and quality (Phred Q ≥ 7).

### 2.7. K-Mer Fingerprinting Pipeline and Consensus Generation

A reference k-mer profile (k = 25) was constructed from a confirmed HEV-positive control (barcode07; wastewater, Antioquia), retaining only k-mers with sequencing depth ≥ 50× to minimize error-derived k-mers. This locally derived reference—rather than an internationally deposited sequence—was a deliberate methodological choice, motivated by the known phylogenetic distinctiveness of Colombian HEV-3 relative to European and Asian GenBank references; using an international reference would risk underestimating HEV signal in samples carrying divergent local strains. Individual reads from all barcodes (abbreviated as bc in tables and figures, e.g., bc02 for barcode02) were scored by the proportion of matching k-mers against this profile, with a ≥5% matching threshold applied to classify reads as HEV-positive. Statistical significance of HEV enrichment was assessed with Fisher’s exact test (one-tailed) against an internal low-signal reference barcode, with 95% Wilson confidence intervals (CIs). K-mer divergence was evaluated across k = 7–25 by calculating profile overlap against GenBank references FJ998008.1 (HEV-3, swine, The Netherlands) and AF060668.1 (HEV-US1, HEV-3, human, USA). Consensus sequences were generated with minimap2 v2.28 (map-ont mode) against FJ998008.1, followed by iVar v1.4.4 (minimum frequency 0.5; minimum depth 10×) on the Galaxy platform (https://usegalaxy.org/). Sequences with >5% International Union of Pure and Applied Chemistry (IUPAC) ambiguity codes were excluded from GenBank deposition.

### 2.8. Inter-Matrix Jaccard Similarity Analysis

Inter-matrix sequence-feature similarity was evaluated using the Jaccard index at k = 25 among the porcine fecal sample (barcode05), the two human clinical samples (barcodes02 and 08), and the environmental wastewater sample (barcode07). The Jaccard index was calculated from the corresponding sets of k-mers as the size of their intersection divided by the size of their union [17]. The analysis quantified the proportion of shared k-mer features between sample profiles. Because barcode07 was also used as the locally calibrated HEV reference profile, this comparison was treated as exploratory and interpreted as sequence-feature similarity rather than as evidence of transmission direction or a direct epidemiological link.

### 2.9. Phylogenetic Analysis

Consensus sequences were aligned against representative GenBank HEV reference sequences (Table S1) using a Needleman–Wunsch algorithm tolerant of IUPAC codes. Phylogenetic reconstruction used a custom Python (v3.10) implementation of the neighbor-joining algorithm [18] with Kimura two-parameter (K2P) distances calculated in NumPy v1.24 [19]. Bootstrap support was estimated to be 1000 replicates. HEV genotype 2 (M74506.1, Mexico) served as the outgroup.

### 2.10. Molecular Cloning and Sanger Sequencing

Amplicons from confirmed positive samples were cloned using the TOPO PCR Cloning Kit (Invitrogen, Thermo Fisher Scientific, Waltham, MA, USA) into the pCR2.1-TOPO vector and transformed into *Escherichia coli* TOP10 cells. Positive colonies were selected by blue-white screening and colony PCR. Plasmid inserts were sequenced using the M13F(-20) universal primer (Macrogen Inc., Seoul, Republic of Korea). Resulting sequences were screened for HEV-specific k-mer motifs (k = 11–25) by sliding-window comparison against FJ998008.1 and AF060668.1, in both orientations, with IUPAC tolerance.

### 2.11. Statistical Analysis

Descriptive statistics were used to summarize HEV detection by sample matrix and municipality, with results reported as proportions. For the sequencing analysis, HEV enrichment was assessed using a one-tailed Fisher’s exact test with 95% Wilson confidence intervals. The inter-matrix Jaccard analysis was descriptive and exploratory; no inferential statistical test was applied to Jaccard values because the comparison involved a small number of molecularly characterized samples.

### 2.12. Generative AI Use

During manuscript preparation, ChatGPT (GPT-4; OpenAI, San Francisco, CA, USA) was used for language editing, structural organization, and formatting support. The tool was not used to generate study data, perform statistical analyses, or determine scientific conclusions. All manuscript content was reviewed and approved by the authors.

## 3. Results

### 3.1. One Health Surveillance: HEV Circulation Across Compartments (2019–2024)

Fifty-four environmental samples and five clinical samples were analyzed across six municipalities in Quindío, including sampling in the Barcelona corregimiento of Calarcá between July 2019 and December 2023. HEV genome was detected in 8/42 pig fecal samples (19.0%), 4/8 runoff water samples (50.0%), and 1/4 tributary water samples (25.0%). Of five serum samples from patients with hepatitis of undetermined etiology, four (80.0%) were RT-qPCR positive for HEV (Table 1 and Table 2). Notably, one HEV-positive patient originated from the same municipality where porcine fecal positivity had been identified.

### 3.2. K-Mer Detection Confirms HEV Signal and Reveals Substantial Sequence Divergence

A k-mer reference profile (k = 25) built from 3380 high-confidence k-mers of the confirmed positive control (barcode07, wastewater, Antioquia) produced statistically significant HEV enrichment in three barcodes. Barcode02 (clinical sample, Quindío) showed 44.5% HEV-classified reads (95% CI: 43.1–45.9%; *p* < 10^−106^), barcode08 (clinical sample, Quindío) showed 39.5% (95% CI: 38.0–41.0%; *p* < 10^−121^), and the environmental control barcode07 showed 97.2%. Five barcodes displayed background signal of 8–15%, classified as negative. The clear bimodal separation between positive (39.5–97.2%) and negative (8–15%) barcodes—with no intermediate cases—supports the specificity of detection.

K-mer overlap analysis across k = 7–25 revealed complete absence of overlap between the Colombian HEV profile and all GenBank reference sequences at k ≥ 17. At k = 25, overlap with HEV-3 references FJ998008.1 and AF060668.1 was 0%, meaning no 25-nucleotide sequence present in the Colombian amplicons exists in any currently deposited HEV sequence. This finding provides a plausible molecular explanation for the difficulty of conventional alignment-based characterization of Colombian HEV sequences: the observed divergence from available international references may reduce alignment sensitivity for short amplicons. Jaccard similarity analysis at k = 7 supported closest affinity with HEV genotype 3 references across all positive barcodes, enabling genogroup-level classification independent of full sequence alignment.

### 3.3. Conserved Diagnostic Motifs Despite Overall Divergence

Despite this substantial divergence, six predefined diagnostic motifs in the ORF2/ORF3 region were detected in the positive samples, with a maximum Hamming distance of 2 mismatches per motif (Table 3). The HE361 primer-binding site was consistently detected across positive barcodes, supporting specific amplification of the HEV ORF2/ORF3 region.

### 3.4. Phylogenetic Placement Within HEV Genotype 3

Phylogenetic analysis of the bc02 consensus (~410 bp ORF2/ORF3 region) against representative sequences from all four human HEV genotypes placed the Colombian isolate unambiguously within the HEV-3 clade (bootstrap support 83%). The expected topology was recovered, with HEV-1 and HEV-4 forming a sister clade (bootstrap 97%) and HEV-2 (M74506.1, Mexico) correctly resolved as the outgroup (bootstrap 63%). K2P genetic distances between the Colombian isolate and HEV-3 references were 4.1% (bc02 vs. FJ998008.1) and 3.6% (bc02 vs. AF060668.1) compared with only 0.7% distance between the two reference sequences themselves.

Figure 2 shows the neighbor-joining phylogenetic tree.

### 3.5. Sanger Confirmation

Sanger sequencing of a cloned amplicon from clinical sample bc08 (Quindío, 2023) produced a 174 bp insert spanning the ORF2/ORF3 junction. K-mer analysis (k = 11–25) identified six HEV-specific motifs with 96–100% identity to reference sequences (Table 3), absent in porcine endogenous retrovirus (PERV), hepatitis A virus (HAV), and hepatitis B virus (HBV) comparator sequences. The motif at positions 150–180 directly matched the ONT bc02 consensus, establishing base-level concordance between both independent sequencing platforms. Figure 3 and Figure 4 illustrate the genome mapping of detected motifs and k-mer distribution across the Sanger insert, respectively.

### 3.6. Inter-Matrix Jaccard Similarity

Inter-matrix Jaccard analysis at k = 25 showed very high sequence-feature similarity between the two human clinical samples (J = 0.90). Human–environmental comparisons showed Jaccard similarity values of 0.65–0.72, whereas human–porcine comparisons ranged from 0.46 to 0.51. The porcine–environmental comparison showed lower similarity (J = 0.33). These values indicate shared sequence features among samples from different matrices, with the highest similarity observed between the two human samples. The analysis was considered exploratory and was not used to infer transmission direction or a direct zoonotic event (Figure 5).

### 3.7. GenBank Deposition: First Colombian HEV-3 Genomic Records

Two sequences were deposited in GenBank, the first Colombian HEV-3 genomic records in an international database. Accession PZ262436 corresponds to the Sanger-confirmed clinical sequence (174 bp, ORF2/ORF3, human serum, Quindío, 2023; isolate HEV-Col-QND-2023-01). Accession PZ262437 corresponds to the ONT wastewater consensus (1366 bp, ORF2 region, Medellín, Antioquia, ~2022; isolate HEV-Col-ANT-2022-01)—the longest Colombian HEV-3 sequence deposited to date.

## 4. Discussion

### 4.1. HEV-3 Circulation Across One Health Compartments

The main epidemiological finding of this study was the detection of HEV RNA across animal, environmental, and human compartments within the same regional surveillance framework in Quindío. These proportions should be interpreted in relation to the different sampling designs and sample sizes rather than as direct estimates of population prevalence. Nevertheless, the concordant detection of viral RNA in animal, environmental, and human compartments is consistent with the One Health framework and extends previous Colombian evidence, which has documented HEV in patients, swine, and environmental waters mainly in Antioquia [4,5,6,7,8,9,10,11].

The relatively high proportion of positive runoff samples is particularly relevant because runoff provides a plausible interface between animal production systems and downstream environmental compartments. However, only eight runoff samples were tested and sampling was distributed across different years and municipalities, so the observed 50.0% positivity should not be interpreted as a generalized prevalence estimate for agricultural runoff in Quindío. Instead, the finding indicates that HEV RNA can be detected in water associated with pig-farming environments and supports the value of including these interfaces in environmental surveillance. The detection of HEV RNA in tributary water, although based on four samples, further suggests that viral material may reach water bodies beyond the immediate farm setting. These observations are compatible with previous Colombian reports of HEV in environmental waters [4] and with the recognized role of fecal contamination and water exposure in HEV transmission [3,4].

The clinical findings should likewise be interpreted cautiously. Four of five patients with hepatitis of undetermined etiology were RT-qPCR positive, but the small clinical sample does not permit estimation of HEV prevalence among patients with hepatitis in the department. The fact that clinical cases were detected alongside porcine and environmental positives in the same regional surveillance framework is nevertheless important because it supports continued investigation of possible links among these compartments. At this stage, the available data establish co-occurrence rather than a common transmission chain.

### 4.2. Genomic Divergence and the Challenge of Reference-Based Detection

A second major finding was the combination of clear HEV-3 classification with marked divergence from the international sequences used as references. The k-mer overlap analysis showed no shared k-mers with the evaluated GenBank references at k ≥ 17, including the HEV-3 references FJ998008.1 and AF060668.1 at k = 25. This result does not establish that the Colombian viruses are globally unique, but it indicates that the short genomic regions analyzed in this study are substantially different from the reference sequences evaluated. The phylogenetic analysis provides an important complement to this observation: despite the large sequence distance, the Colombian consensus clustered within the HEV-3 clade with 83% bootstrap support.

This combination helps explain why conventional reference-dependent approaches may perform poorly when local sequence diversity is not represented in the reference set. Colombian studies have previously generated molecular evidence for HEV-3 using comparison with GenBank sequences [5,10,11], but the increasing accumulation of evidence from different biological compartments makes the composition of reference databases increasingly relevant to surveillance. Our results therefore support the use of local reference material as a complement to, rather than a replacement for, international sequence databases. The present data also argue against interpreting a failure to obtain a conventional alignment as evidence that HEV is absent. Instead, reference mismatch should be considered among the possible explanations when laboratory evidence points toward infection but sequence similarity is weak.

### 4.3. Concordance of Independent Molecular Evidence

The molecular characterization was strengthened by agreement among several independent approaches. The locally calibrated k-mer fingerprinting identified strong HEV signal in the two clinical barcodes, with substantial enrichment over background. Diagnostic k-mer motifs were then detected in the ORF2/ORF3 region, including sequences spanning the capsid and ORF2/ORF3 junction. The independent Sanger sequence reproduced the diagnostic motifs and showed direct sequence concordance with the ONT consensus in the downstream region. Finally, the phylogenetic analysis placed the Colombian sequence within HEV-3. Each method addresses a different source of uncertainty: k-mer profiling detects related sequence features without requiring close whole-read alignment, motif analysis tests local sequence features, Sanger provides base-level confirmation, and phylogenetic reconstruction establishes the genotype context.

This convergence is especially relevant for a surveillance setting in which the available reference sequences may not capture local diversity. It also reduces the likelihood that the HEV-3 classification is explained solely by one analytical method or by a single database-dependent signal. At the same time, the sequencing strategy was limited to an amplicon in the ORF2/ORF3 region, so the data do not support whole-genome comparison or subgenotype assignment. The appropriate interpretation is therefore regional genomic confirmation of HEV-3 rather than complete genomic characterization of the circulating viral population.

### 4.4. Inter-Matrix Jaccard Similarity and Epidemiological Interpretation

The inter-matrix Jaccard analysis provides a complementary view of sequence-feature similarity across the porcine, human, and environmental samples. The two human samples showed the greatest similarity (J = 0.90), while human-environmental comparisons ranged from 0.65 to 0.72. Human–porcine comparisons were lower (0.46–0.51), and the porcine-environmental comparison was lower still (J = 0.33). These values suggest that related k-mer features were shared among samples from different matrices, but the analysis cannot determine whether one matrix is the source of another. In particular, a higher human-environmental Jaccard value should not be interpreted as evidence that environmental HEV originated from a human infection, nor does the lower porcine-environmental value exclude an animal contribution.

The Jaccard results are best considered hypothesis-generating and should be interpreted together with the RT-qPCR, phylogenetic, and Sanger findings. An additional methodological consideration is that barcode07 was used to construct the locally calibrated HEV k-mer profile and was also included in the inter-matrix comparison. This creates a degree of dependence that makes the Jaccard values unsuitable for independent epidemiological inference. For that reason, the analysis is useful as an exploratory description of shared sequence features, but stronger conclusions about environmental circulation or zoonotic transmission would require independent environmental samples, additional animal and human sequences, and ideally temporal or spatial linkage between the compartments.

### 4.5. Implications for One Health HEV Surveillance in Colombia

Taken together, the findings support an expanded One Health approach to HEV surveillance in Colombia. The detection of HEV RNA in porcine feces and farm-associated waters, together with clinical positives and genomic confirmation of HEV-3, indicates that surveillance restricted to symptomatic patients is unlikely to capture the full epidemiological context of HEV. Environmental sampling can identify viral circulation at interfaces where animal waste, water, and agricultural activity overlap, while genomic characterization can help determine whether sequences detected in different settings are related.

The results also highlight a practical need for regional reference sequences. The difficulty of matching the Colombian profiles to the international references evaluated here suggests that sequence databases should represent the diversity present in Latin America rather than relying predominantly on isolates from other geographic regions. Future work should include attempts at viral isolation in cell culture, which would enable phenotypic characterization and facilitate antiviral testing. Public deposition of the sequences generated in this study provides a small but useful contribution to that objective. Future work should expand the number of sequenced samples, include complete or near-complete genomes when feasible, and integrate molecular results with information on farm location, water flow, animal management, food exposure, and patient history. Such data would allow the exploratory associations observed here to be tested with stronger epidemiological designs.

### 4.6. Limitations

Several limitations should be considered. First, the study was observational and sampling was uneven across municipalities and years, limiting direct comparisons of detection frequencies between locations and matrices. Second, the number of clinical and molecularly characterized samples was small, which restricts conclusions about prevalence and transmission. Third, sequencing targeted a single genomic region and therefore does not provide whole-genome diversity or robust subgenotype resolution. Fourth, the porcine origin assigned to barcode05 was inferred from co-detection of porcine *Kobuvirus* as a proxy marker and should be confirmed against laboratory records. Fifth, the Jaccard analysis was exploratory, involved a small number of samples, and included barcode07 as both the local k-mer reference and one of the compared environmental samples. Sixth, serological testing (IgM/IgG anti-HEV) was not performed on the clinical samples, which prevents distinguishing between acute and past infections and precludes seroprevalence estimation in this cohort. These limitations do not negate the molecular evidence for HEV-3, but they define the appropriate level of epidemiological inference from the present study.

## 5. Conclusions

This study demonstrates HEV-3 detection across environmental, porcine, and human compartments in Quindío, Colombia (2019–2024). The molecular findings are compatible with a possible zoonotic connection but do not establish transmission direction or a direct animal-to-human transmission event. The study further reports the first genomic characterization and GenBank deposition of Colombian HEV-3 sequences identified by this investigation and highlights substantial sequence divergence from the available international references. The locally calibrated k-mer fingerprinting pipeline, combined with Oxford Nanopore amplicon sequencing, Sanger validation, phylogenetic analysis, and exploratory inter-matrix similarity analysis, provides a potentially useful framework for HEV-3 surveillance in settings where reference databases may incompletely represent local viral diversity. These findings support expanded genomic surveillance and the inclusion of regional reference sequences in pathogen monitoring frameworks.

## Figures and Tables

**Figure 1:**
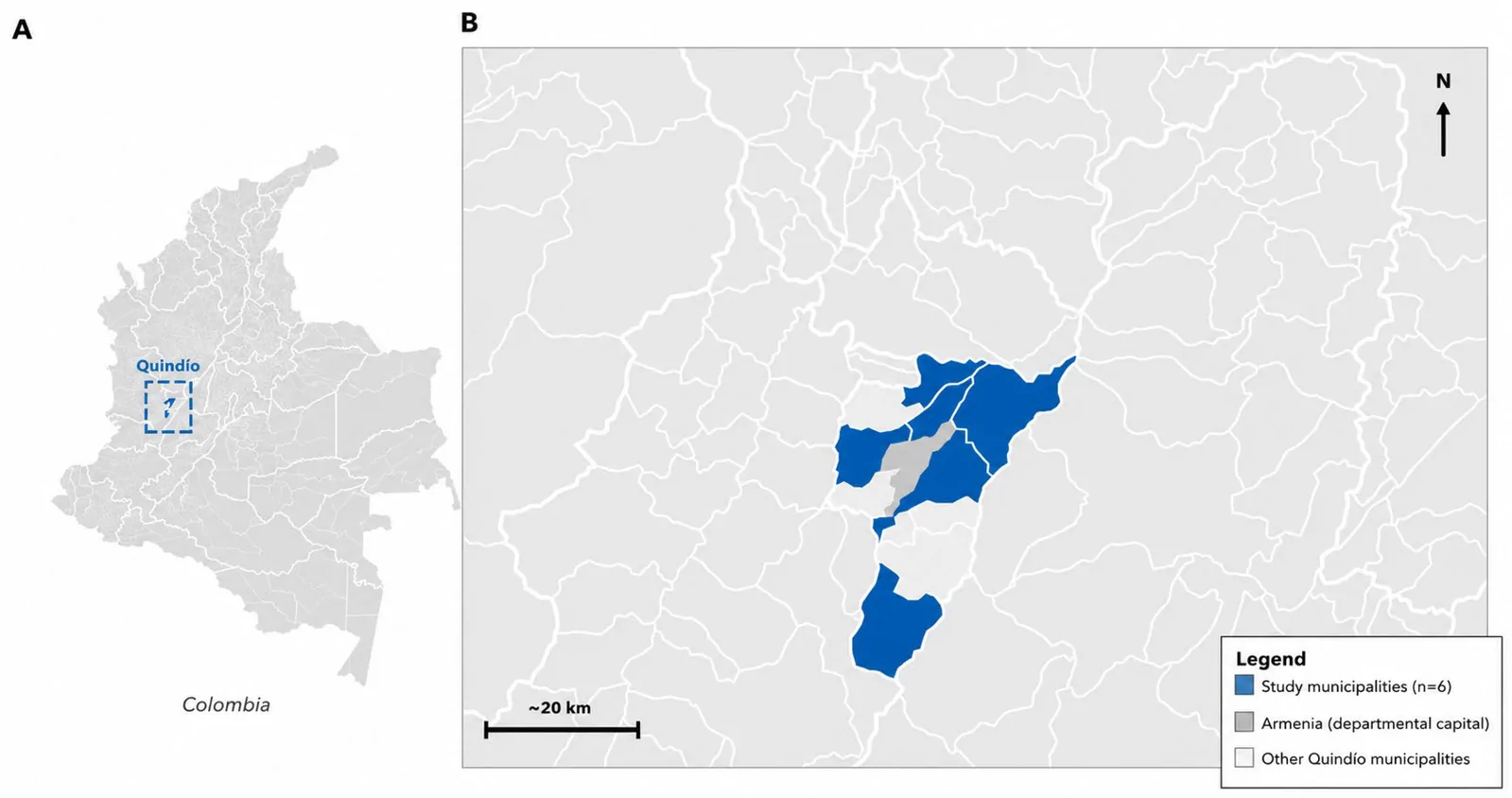
Location of the study area. (**A**) Location of Quindío Department within Colombia (dashed box). (**B**) Quindío Department, showing the six study municipalities (blue) where fecal, runoff water, and tributary water samples were collected, and Armenia, the departmental capital (gray). The Barcelona corregimiento is located within the municipality of Calarcá.

**Figure 2:**
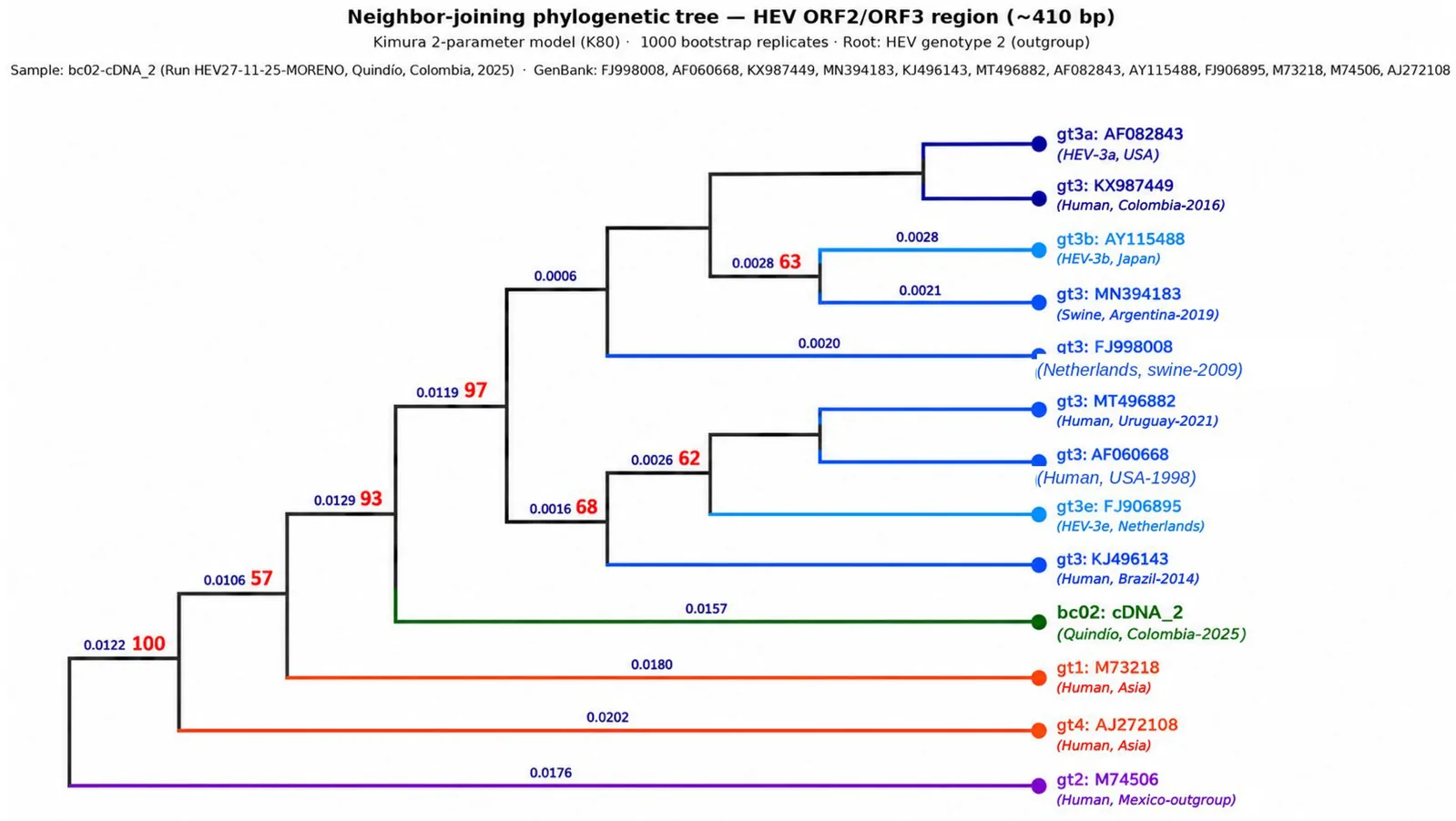
Neighbor-joining phylogenetic tree based on Kimura two-parameter distances (1000 bootstrap replicates). Bootstrap values ≥ 60% are shown at nodes. Colombian isolates (bc02, Quindío) are shown in bold within the HEV-3 clade (bootstrap 83%). Outgroup: HEV-2 (M74506.1, Mexico). Scale bar indicates nucleotide substitutions per site. gt, genotype; K80, Kimura two-parameter model.

**Figure 3:**
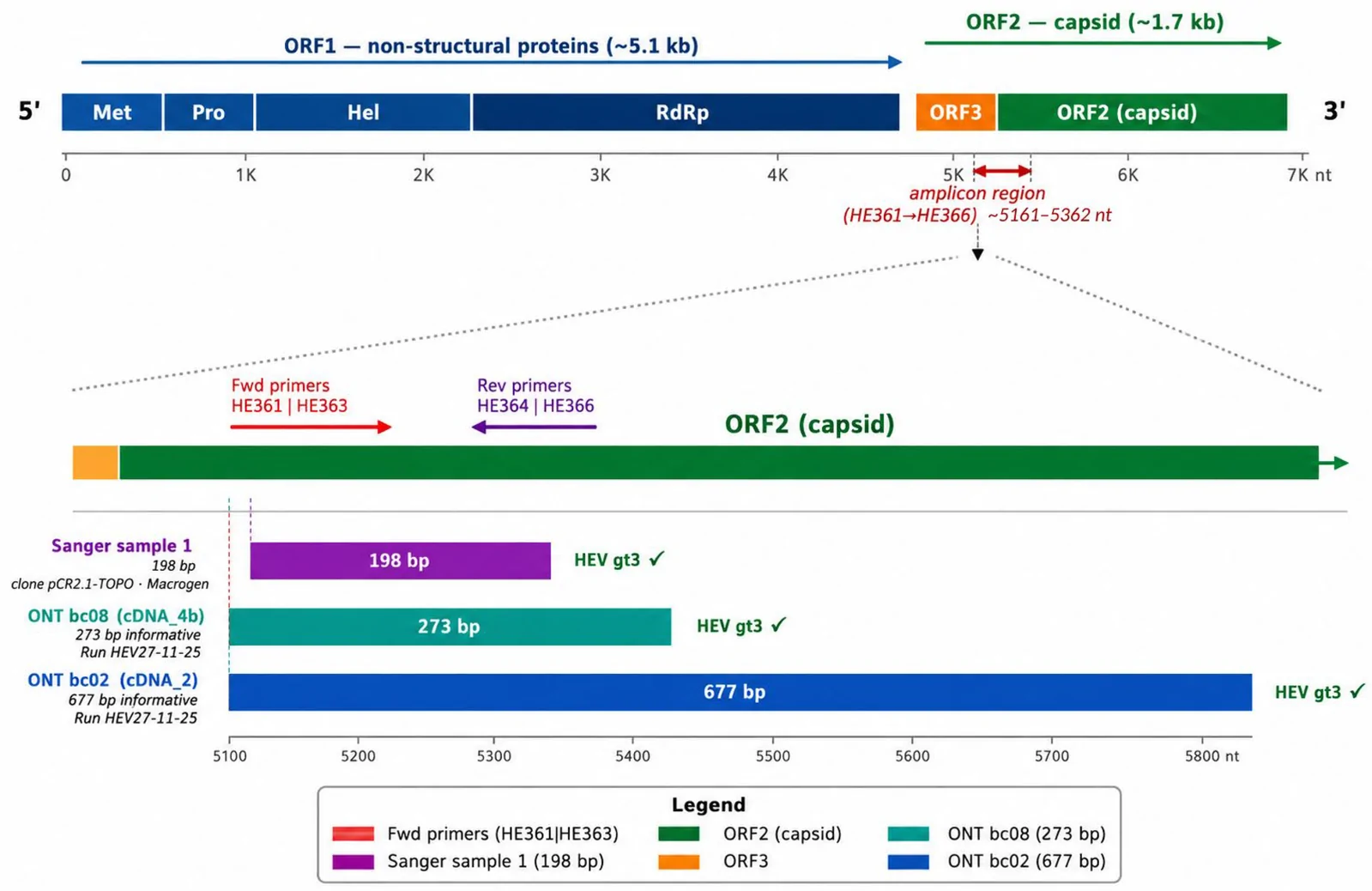
Schematic map of the HEV-3 genome (~7200 bp; reference FJ998008.1) indicating the location of the ORF2/ORF3 amplicon region (primers HE361/HE366, positions ~5235–5462) and the six diagnostic k-mer motifs identified in the Sanger sequence. Colored arrows denote individual motif positions. Gray dotted lines connect the amplicon region on the genome map to the expanded view below; vertical dashed lines mark the amplicon boundaries and the start positions of the aligned sequences. Met, methyltransferase; Pro, protease; Hel, helicase; RdRp, RNA-dependent RNA polymerase; Fwd, forward; Rev, reverse; gt, genotype; nt, nucleotides.

**Figure 4:**
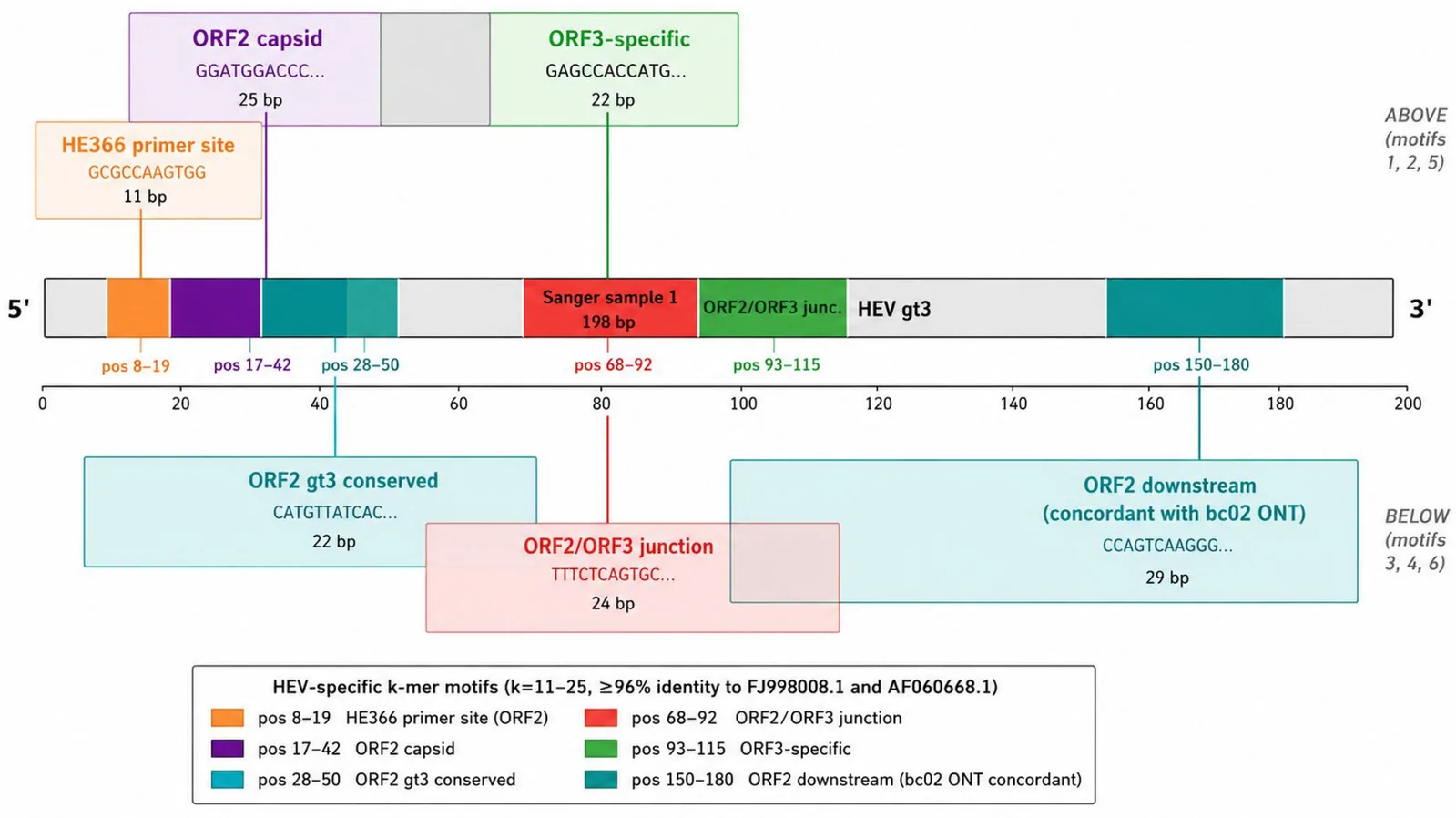
K-mer fingerprinting map of the 174 bp Sanger insert (accession PZ262436, Quindío 2023). Colored blocks represent HEV-specific diagnostic k-mers (k = 11–25) with ≥96% identity to FJ998008.1 and AF060668.1. Positions 150–180 show direct sequence overlap with the ONT bc02 consensus. Motif sequences truncated with ellipses (…) in the figure are given in full in Table 3. pos, position; junc., junction; gt, genotype.

**Figure 5:**
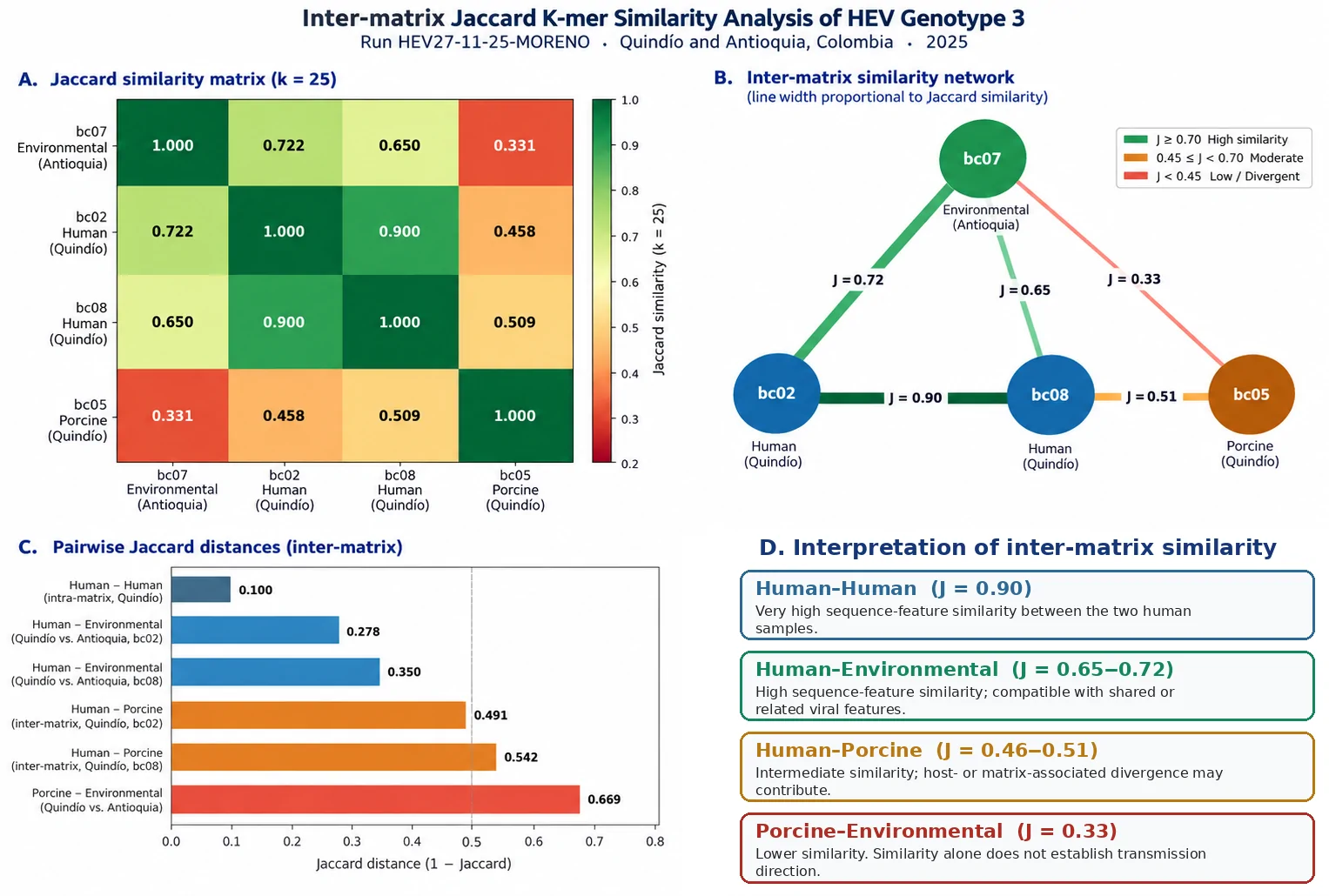
Inter-matrix Jaccard k-mer similarity analysis (k = 25) across porcine fecal (bc05), human clinical (bc02, bc08), and environmental wastewater (bc07) samples from run HEV27-11-25-MORENO. Panel (**A**): Similarity matrix. Panel (**B**): Inter-matrix similarity network. Panel (**C**): Pairwise Jaccard distances, the vertical dashed line indicates a Jaccard distance of 0.5 (Jaccard similarity of 0.5) and is shown as a visual reference. Panel (**D**): Interpretation of inter-matrix similarity. The observed similarities describe shared sequence features across matrices but do not establish transmission direction or a direct zoonotic link. Because bc07 was used as the locally calibrated k-mer reference profile, the inter-matrix comparison was interpreted as exploratory. J, Jaccard index.

**Table 1:** Environmental and porcine samples by municipality and RT-qPCR result, Quindío department, 2019–2023.

HEV+ Tributary	HEV+ Runoff	HEV+ Fecal	Tributary (*n*)	Runoff (*n*)	Pig Fecal (*n*)	Municipality	Year
—	1	3	—	1	10	Filandia	2019
—	—	—	1	1	4	Salento	
—	—	—	—	1	2	Montenegro	
—	—	—	—	—	3	Calarcá	
—	1	1	1	1	1	Circasia	2021
—	1	—	1	1	1	Calarcá	
—	—	1	—	1	2	Filandia	
—	1	—	—	1	1	Circasia	2022
1	—	1	1	1	2	Génova	
—	—	—	—	—	11	Filandia	
—	—	1	—	—	4	Calarcá	
—	—	1	—	—	3	Calarcá (Barcelona)	
—	—	—	—	—	1	Calarcá	2023
1 (25.0%)	4 (50.0%)	8 (19.0%)	4	8	42	6 municipalities	TOTAL

Note. HEV+ = samples positive by RT-qPCR (MIKROGEN^®^ ampliCube HEV 2.0 Quant). Em dashes (—) indicate no samples collected or no positives detected. Barcelona is a corregimiento of the municipality of Calarcá and is therefore included within the six municipalities represented in Figure 1.

**Table 2:** Clinical serum samples from patients with hepatitis of undetermined etiology, Quindío, 2022–2024.

Clinical Outcome	Clinical Diagnosis at Admission	RT-qPCR Result	Age (Years)	Sex	Municipality	Sample ID
Self-limited hepatitis	Abdominal pain is under evaluation. Icteric syndrome under evaluation	Positive	40	Female	Calarcá (rural area)	bc02/cDNA-2
Self-limited hepatitis	Icteric syndrome under evaluation	Positive	35	Female	Armenia (urban)	bc08/cDNA-4b
Deceased (ICU (intensive care unit))	Multiple organ failure.	Positive	36	Female	Armenia (homeless)	bc01/cDNA-1
Deceased	Icteric syndrome under evaluation	Positive	50	Male	Calarcá (rural area)	Serum-4
Self-limited hepatitis	Icteric syndrome under evaluation	Negative	28	Male	Armenia Healthcare worker	Serum-5

**Table 3:** Diagnostic k-mer motifs of HEV genotype 3 identified in the 174 bp Sanger-derived ORF2/ORF3 sequence from a clinical sample from Quindío, Colombia. Motifs were identified by sliding-window k-mer analysis (k = 11–25) and compared with HEV-3 reference sequences FJ998008.1 and AF060668.1.

Identity to FJ998008.1	Genomic Domain	Length	Position (bp)	Motif (k-mer)
100%	HE366 primer site—ORF2	11 bp	8–19	GCGCCAAGTGG
100%	ORF2 capsid—HEV-specific	25 bp	17–42	GGATGGACCCAGGTCATGTTATCAC
100%	ORF2 conserved in HEV-3	22 bp	28–50	CATGTTATCACTCCTCTCTGGG
100%	ORF2/ORF3 overlap junction	24 bp	68–92	TTTCTCAGTGCAAGCAAAACGAAG
96%	ORF3—HEV-specific	22 bp	93–115	GAGCCACCATGATCTCCCGCCC
100%	ORF2 downstream/bc02 ONT concordance	29 bp	150–180	CCAGTCAAGGGCGAATTCCAGCACACTGG

## Data Availability

The sequences generated in this study have been submitted to NCBI GenBank (Submission ID 3072924, isolate HEV-Col-QND-2023-01, accession PZ262436; Submission ID 3072948, isolate HEV-Col-ANT-2022-01, accession PZ262437). Both accession numbers are publicly available in GenBank as of July 2026. Raw sequencing data and analysis scripts are available from the corresponding author upon reasonable request.

## Supplementary Materials

The following supporting information can be downloaded at: https://www.mdpi.com/article/10.3390/tropicalmed11100290/s1, Table S1: GenBank reference sequences used in the phylogenetic analysis.
