# Supplementary material for: Hepatitis E Virus Genotype 3 in Quindío, Colombia: One Health Surveillance and First Genomic Characterization Reveal Cryptic Regional Divergence
Source: Trop Med Infect Dis. 2026 Oct 13;11(10):290. doi: 10.3390/tropicalmed11100290 (PMC13709530; doi:10.3390/tropicalmed11100290)
Supplement: Supplementary file 1 [file tropicalmed-11-00290-s001.zip › tropicalmed-4588635-supplementary.pdf]

**Supplementary Materials: Hepatitis E Virus Genotype 3 in Quindío, Colombia:**

**One Health Surveillance and First Genomic Characterization Reveal Cryptic Regional Divergence**

**Table S1.** GenBank reference sequences used in the phylogenetic analysis. Sequences were aligned with Colombian HEV-3 consensus sequences (bc02 and bc07) using the Needleman–Wunsch algorithm with IUPAC ambiguity code tolerance. Phylogenetic reconstruction was performed using a custom neighbor-joining (NJ) implementation with Kimura 2-parameter (K2P) distances and 1,000 bootstrap replicates. HEV genotype 2 (M74506.1, Mexico) was used as outgroup. Study sequences deposited in GenBank are indicated with †.

| GenBank Accession | HEV Genotype | Strain / Description | Host | Country | Year Deposited | Role in Analysis |
|---|---|---|---|---|---|---|
| FJ998008.1 | HEV-3 | Swine HEV, Netherlands (swine feces, genotype 3f) | Swine | Netherlands | 2009 | HEV-3 reference; primary mapping reference for minimap2/ivar consensus generation; K2P distance 4.1% vs. bc02 |
| AF060668.1 | HEV-3 | HEV-US1 (human hepatitis E, genotype 3a) | Human | USA | 1999 | HEV-3 reference (subgenotype 3a); K2P distance 3.6% vs. bc02; k-mer identity analysis |
| AB074915.3 | HEV-3 | HEV genotype 3, strain JAK-Sai (swine) | Swine | Japan | 2003 | HEV-3 reference (phylogenetic tree); 95% similarity to Antioquia porcine strains (Gutiérrez-Vergara et al. 2015) |
| LR777865.1 | HEV-3 | Paslahepevirus balayani isolate (genotype 3) | Human | United Kingdom | 2016 | HEV-3 reference (phylogenetic tree); used to represent European HEV-3 clade |
| M73218.1 | HEV-1 | HEV genotype 1a (Burma strain; Tam et al. 1991) | Human | Myanmar | 1992 | HEV-1 reference; sister clade with HEV-4 (bootstrap 97%); outgroup in earlier UPGMA analysis |

| | | | | | | |
|---|---|---|---|---|---|---|
| **AJ272108.1** | HEV-4 | HEV genotype 4 (swine, China) | Swine | China | 2001 | HEV-4 reference; sister clade with HEV-1 (bootstrap 97%) |
| **M74506.1** | HEV-2 | HEV genotype 2 (Sar-55 strain; Pakistan/Mexico lineage) | Human | Mexico | 1992 | Outgroup for NJ phylogenetic reconstruction (bootstrap 63%) |
| **MN646691.1** | HEV-3 | HEV clone HEPAC-12-3, complete genome | Swine | France / United Kingdom | 2020 | BLAST top hit for bc07 (100% identity / 21 bp); HEV-3, European porcine lineage |
| **MN646690.1** | HEV-3 | HEV clone HEPAC-12-2, complete genome | Swine | France / United Kingdom | 2020 | BLAST top hit for bc07 (100% identity / 21 bp); HEV-3, European porcine lineage |
| **PZ262436 †** | HEV-3 | HEV-Col-QND-2023-01; 174 bp, ORF2/ORF3 partial; Sanger sequencing | Human | Colombia (Quindío) | 2025 | Study sequence — clinical sample bc08/Muestra 1; first Colombian HEV-3 Sanger sequence in GenBank |
| **PZ262437 †** | HEV-3 | HEV-Col-ANT-2022-01; 1,366 bp, ORF2 region; Oxford Nanopore (ONT) | Environmental (wastewater) | Colombia (Antioquia) | 2025 | Study sequence — wastewater sample bc07; longest Colombian HEV-3 sequence in GenBank |

† *Sequences generated in the present study and deposited in GenBank (BankIt Submission IDs: 3072924 and 3072948). Accession numbers PZ262436 and PZ262437 assigned by NCBI GenBank.*

**Abbreviations:** *NJ, neighbor-joining; K2P, Kimura 2-parameter; bp, base pairs; ORF, open reading frame; ONT, Oxford Nanopore Technology; BLAST, Basic Local Alignment Search Tool.*

**K2P genetic distances (bc02 vs. reference sequences):** *bc02 vs. FJ998008.1 = 4.1%; bc02 vs. AF060668.1 = 3.6%; FJ998008.1 vs. AF060668.1 = 0.7%. Bootstrap values: HEV-3 clade (bc02) = 83%; HEV-1/HEV-4 sister clade = 97%; outgroup (M74506.1) = 63%.*
